# Synthesis of Hybrid-Polypeptides *m*-PEO-*b*-poly(His-*co*-Gly) and *m*-PEO-*b*-poly(His-*co*-Ala) and Study of Their Structure and Aggregation. Influence of Hydrophobic Copolypeptides on the Properties of Poly(L-histidine)

**DOI:** 10.3390/polym9110564

**Published:** 2017-10-30

**Authors:** Dimitrios Skoulas, Dimitra Stavroulaki, Konstantinos Santorinaios, Hermis Iatrou

**Affiliations:** Department of Chemistry, University of Athens, Panepistimiopolis, 15771 Zografou, Athens, Greece; dskoulas@chem.uoa.gr (D.S.); stavrulakidim@yahoo.gr (D.S.); santor.g7@gmail.com (K.S.)

**Keywords:** polypeptides, Ring-Opening Polymerization (ROP), *N*-carboxyanhydrides (ΝCAs), Poly(L-histidine) (PHis), nanoparticles

## Abstract

The highly diverse and sophisticated action of proteins results from their equally diverse primary structure, which along with the nature of interactions between the amino acids, defines the higher self-assembly of proteins. The interactions between amino acids can be very complicated, and their understanding is necessary in order to elucidate the protein structure-properties relationship. A series of well-defined hybrid-polypeptidic diblock copolymers of the type *m*-PEO-*b*-poly(His-*co*-Gly) and *m*-PEO-*b*-poly(His-*co*-Ala) was synthesized through the ring opening polymerization of the *N*-carboxyanhydrides of the corresponding amino acids, with a molar ratio of the hydrophobic peptide to histidine at 10%, 20% and 40%. The excellent purity of the monomers combined with the high vacuum techniques resulted in controlled polymerization with high molecular and compositional homogeneity. FT-IR, as well as circular dichroism, were employed to investigate the secondary structure of the polymers, while DLS, SLS and ζ-potential were utilized to study the aggregates formed in aqueous solutions, as well as their pH responsiveness. The results revealed that the randomly distributed monomeric units of glycine or alanine significantly influence L-histidine’s structure. Depending on the pH, aggregates with a different structure, different molecular characteristics and a different surface charge are formed, potentially leading to very interesting bioapplications.

## 1. Introduction

Synthetic polypeptides synthesized via controlled polymerization exhibit complex compositions and architectures and have been investigated over recent decades as a promising system for many bio-applications [1,2,3]. The advantages of these polypeptides include biodegradability, biocompatibility, inherent active residue groups, their modification with other chemical moieties and their native secondary structures. Drug and gene delivery systems based on polypeptides [4] that can respond to external stimuli, e.g., pH, light, redox, temperature, and enzymes, have drawn great attention for their potential biological applications [5]. These systems can self-assemble into aggregates such as micelles [6], polymersomes [7], nanocapsules [8], hydrogels [9], and nanogels [10] and can selectively deliver a cargo with therapeutic properties to specific tissues, cells or organs. Due to their ability to alter conformational structure and change physical or chemical properties under the influence of external stimuli, these systems are often referred as “stimuli-responsive” [11]. 

In order to exploit polypeptide characteristics, the predetermination of their properties by adopting the appropriate macromolecular architecture, molecular weights and composition are necessary, and in order to make these systems functional, control of their molecular characteristics is of key importance. The most commonly used method for the synthesis of block copolypeptides is the ring opening polymerization (ROP) of *N*-carboxyanhydrides (ΝCAs) [12]. The ROP of NCAs offers a plethora of benefits, since it affords well-defined amphiphilic polypeptide materials [13,14]. In addition, the covalent attachment of poly(ethylene oxide) (PEO or PEG) to polypeptides could result in the formation of aggregates with “stealth” and protective properties when PEG is in the outer periphery, since the hydrophilic and flexible PEG chains form a shell that prevents the interactions with macrophages, due to its spatial conformations [15]. The PEG-modified polypeptides present steric hindrance because of the water that binds to PEG moieties rendering it extended and “bulky” [16]. Hydrogen bonding between the ether oxygen of PEG and water leads to a hydrated film enhancing repulsion between proteins and increasing the hydrodynamic radius of the nanoparticle. Consequently, PEG binding with polypeptides results in increased blood circulation time of the formed nanoparticles, which is one of the requirements for a sustained and prolonged release of their encapsulated cargo [17,18]. Furthermore, the conjugation of PEG to polypeptides corroborates the tolerance against enzymes, improves water solubility and reduces toxicity [19].

Among the polypeptides that have been used for block polymerization with PEG, Poly(L-histidine) (PHis) has unique characteristics which render it a suitable material for biological applications. The amphoteric nature of PHis, due to its unsaturated nitrogen at its imidazole ring, can turn it from soluble at pH 6.3 to insoluble at pH 7.4 [20], thus rendering it a pH stimuli-responsive polypeptide. The ability to form Au nanoparticles [21,22] and to bind with metals including Cu, Fe, Mn, Zn, Co, and Ni has extended its uses [23]. Additionally, PHis can condense DNA and can form polyion complexes through electrostatic interactions [24]. PHis is susceptible to racemization, and the choice for its protecting group is crucial in order to avoid this problem [24]. Trityl protecting group (Trt) is the appropriate protective group to overcome this difficulty [25].

The random distribution of monomeric units of hydrophobic peptides along the PHis chain can alter its secondary structure and its degree of protonation resulting in different solubilities and aggregation properties for the formation of nanoparticles. These possible variations can be exploited through the design of systems with controllable secondary structures and self-assembly ability. The type and the ratio of the randomly distributed hydrophobic units within PHis are expected to lead to systems with different self-assembly properties [25]. Kim et al. have designed poly(ethylene glycol)-*b*-poly(His-*co*-phenylalanine) polymers that formed micelles in aqueous solutions. They reported that the copolymer p*K*_a_ value can be controlled by tuning the histidine to phenylalanine molar ratio in the copolypeptide block [26]. Two other types of hydrophobic peptides that can be examined for this purpose are glycine (Gly) and alanine (Ala). Poly(glycine) has not been widely used or studied. Gaspard et al. [27] have synthesized diblock polypeptides of the type poly(L-lysine)-*b*-polyglycine to study their supramolecular assembly and found that poly(glycine) offers flexibility and compliance to the formulated vesicles. Papadopoulos et al. [28] also studied the self-assembly in a series of poly(*γ*-benzyl-L-glutamate)-*b*-polyglycine (PBLG-*b*-PGly) block copolypeptides. They found that due to the multiple chain folding of PGly, the lamellar structure of PBLG was stabilized. Finally, Yu et al. [29] used poly(glycine) for synthesizing a polymeric tumor-penetrating agent. 

Poly(L-alanine) has been widely used to form hydrogels that present reverse thermal gelation properties [30,31]. In the majority of these systems, by raising the temperature, a sol-to-gel transition occurs [32,33]. The participation of alanine residues mainly confers *α*-helix and *β*-sheet [34,35] secondary structures to these polypeptides. Joo et al. have reported that *β*-sheet conformation was high for the l-alanine polymers while *α*-helix was high for the polymers with the d-isomer [36]. Additionally, the preference that the enzyme elastase shows to alanine has been utilized in several systems for the enzymatic degradation of the polymers containing alanine monomeric units [37]. 

**Scheme 1:**
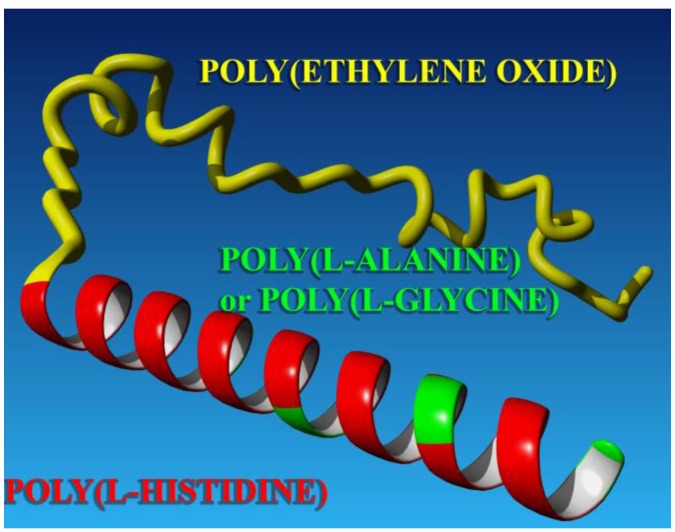
The polymers synthesized: PEO-*b*-P(His-*co*-Ala), PEO-*b*-P(His-*co*-Gly).

Herein, the synthesis of a series of polymers of the type *m*-PEO-*b*-poly(His-*co*-Gly) and *m*-PEO-*b*-poly(His-*co*-Ala) with hydrophobic peptide (Gly or Ala) to histidine peptide molar ratios of 10%, 20%, and 40% is presented (Scheme 1). Trt protecting group was chosen for histidine residues, as it can be easily removed under mild conditions [38] without causing racemization. A combination of different characterization techniques was employed to determine the molecular characteristics of the polymers. Polymer aggregation together with secondary structure and their dependence on pH and temperature were examined by circular dichroism (CD), dynamic light scattering (DLS), static light scattering (SLS) and zeta potential (ζ-potential).

## 2. Materials and Methods

### 2.1. Materials

Boc-His(Trt)-OH (>99%) was purchased from Christof Senn Laboratories AG (Dielsdorf, Switzerland). Triphosgene (99%) was acquired from Acros Organics (Waltham, MA, USA). Triethylamine (>99%, Acros Organics) was dried over calcium hydride for one day and then distilled and stored over sodium. Then, the desired amount was distilled to other apparatus prior to use. *N*,*N*-Dimethylformamide (DMF) was supplied from Fisher Scientific (Waltham, MA, USA) (99.91%), special grade for peptide synthesis with less than 50 ppm active impurities and was further purified by short-path fractional distillation under high vacuum. The middle fraction was always used. Trifluoroacetic acid (≥99.5%, TFA) was also purchased from Fischer Scientific. Thionyl chloride (SOCl_2_) (99.7%, distilled before use) was obtained from Acros Organics. Triethylsilane (99%), Limonene (97%) and diethyl ether (≥99%) were purchased from Aldrich. Purification of benzene (99%, thiophene-free grade, Sigma-Aldrich (Saint Louis, MO, USA), dichloromethane (anhydrous, ≥99.8%, Sigma-Aldrich) and tetrahydrofuran (THF, max 0.005% water, Merck Millipore (Darmstadt, Germany)) was performed using standard high vacuum techniques reported elsewhere [39]. Acetonitrile (Aldrich, 99.9%) was dried over phosphorus pentoxide and then fractionally distilled prior to use. Glycine (>99%) and L-alanine (>99%) were acquired from Aldrich. Ethyl acetate purchased from Merck Millipore (>99.5%) was fractionally distilled over phosphorous pentoxide. Hexane (>99%, Merck Millipore) was fractionally distilled over sodium and afterward distilled over *n*-BuLi. Distilled water was further purified by a Milli-Q water purification system (18.2 MΩ.cm, Merck Millipore). End-functionalized amine-terminated methoxy protected poly(ethylene oxide) (*m-PEO*-NH_2_) having molecular weights of 9.95 × 10^3^ g/mol were purchased from Aldrich and were employed as monofunctional macroinitiators.

### 2.2. Synthesis of Nim-Trityl Protected N-Carboxyanhydride of L-Histidine (Trt-His-NCA)

The synthesis of Trt-His-NCA was accomplished in two steps and has been reported elsewhere in detail [25]. Briefly, in the first step, 10 g (20.1 mmol) of Boc-His(Trt)-OH was dried overnight under high vacuum. The next day, 80 mL of THF were distilled in the flask. The reaction flask was set in an ice-bath, filled with argon, and 1.63 mL (22.4 mmol) of thionyl chloride diluted in 10 mL of THF were added dropwise. After 2 h, diethyl ether was used for precipitation of Trt-His-NCA·HCl. After filtration, 150 mL of ethyl acetate were distilled, and the mixture was placed in a water bath, at 45 °C for 1 h. It was then cooled at 0 °C and Trt-His-NCA·HCl was collected by filtration (6.25 g, 14 mmol). In the second step, 100 mL of ethyl acetate was distilled and the suspension was placed in an ice bath. A solution of 20 mL ethyl acetate and triethylamine (1.75 mL, 12.5 mmol) was slowly added dropwise under vigorous stirring. The resulting triethylamine hydrochloride was filtered off, and recrystallization with hexane took place. A second recrystallization was carried out with a solvent/nonsolvent ethyl acetate/hexane (1:5) mixture, followed by isolation of the white solid by filtration. Finally, Trt-His-NCA was dried under vacuum overnight (5.53 g, 13.0 mmol, 67% yield).

### 2.3. Synthesis of N-Carboxy Anhydride of Glycine (Gly-NCA)

The NCA of glycine (Gly-NCA) was synthesized from the corresponding *α*-amino acid (5.0 g, 66.6 mmol), which was dissolved in acetonitrile. Limonene (6 mL) and triphosgene 7,32 g (24.6 mmol) were added at 70 °C under an inert atmosphere. After filtration, the filtrate was recrystallized by hot filtration. Finally, the NCA was dried under vacuum overnight (1.2 g, 11.8 mmol, 59% yield).

### 2.4. Synthesis of N-Carboxy Anhydride of L-Alanine (Ala-NCA)

The synthesis of the alanine NCA (Ala-NCA) was carried out following to a previously published method [40]. l-alanine 5.0 g (56.1 mmol) was suspended in acetonitrile and triphosgene 6.16 g (20.7 mmol) was added at 70 °C. After filtration, Ala-NCA was then dissolved and dried several times with ethyl acetate under high vacuum, in order to remove the excess triphosgene. Finally, Ala-NCA was recrystallized in a system of ethyl acetate and hexane three times under high vacuum. The purified NCA (1.5 g, 13 mmol, 57% yield) was stored under an inert atmosphere at 0 °C.

### 2.5. Polymer Synthesis

**Scheme 2:**
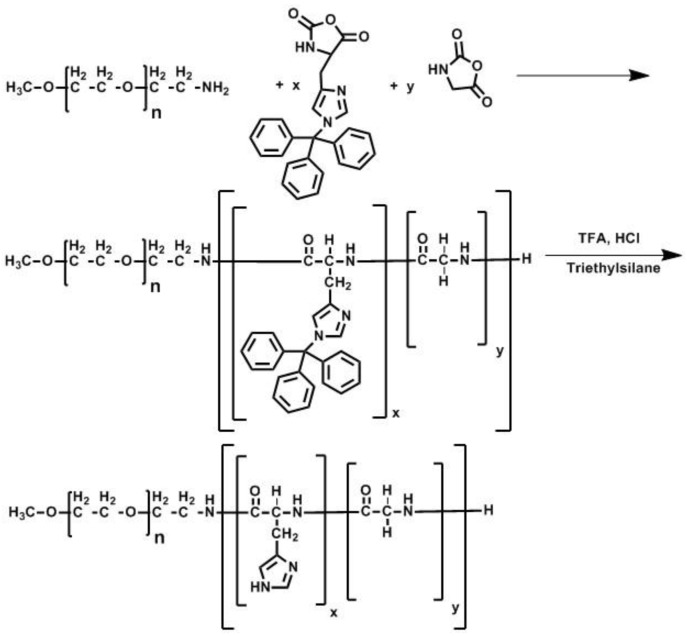
Synthetic route toward the terpolymer *m*-PEO-*b*-poly(His-*co*-Gly).

**Scheme 3:**
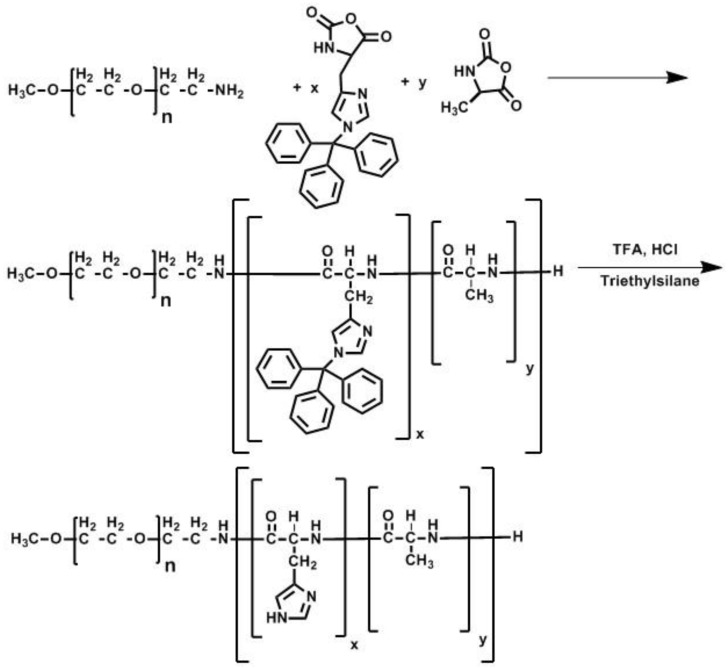
Synthetic route toward the terpolymer *m*-PEO-*b*-poly(His-*co*-Ala).

All manipulations and polymer synthetic procedures were performed under high vacuum in custom-made glass reactors, equipped with break-seals, high vacuum stopcock, glass-covered magnets and constrictions for the addition of reagents under the guidelines of the high vacuum techniques [39]. The reactions involved in the synthesis of the polymers are shown in Scheme 2 and Scheme 3.

A custom-made glass reactor was initially attached to the vacuum line through the ground joint and was evacuated and flame dried several times. It was subsequently inserted into the glove box and 0.2 g *m*-PEO-NH_2_ with molecular weight 9.95 × 10^3^ g/mol (0.02 mmol) were added into the apparatus. This amount of the macroinitiator (0.20 gr, 0.020 mmol –NH_2_) was kept constant for each of the six synthesized polymers. The custom-made glass reactor was connected again to the vacuum line, it was evacuated and the apparatus was remained connected overnight. The next day, 20 mL of dry benzene were distilled and the macroinitiator was dissolved. The solution was stirred for two hours and benzene was distilled off to dryness. DMF (20 mL) was distilled followed by dissolution of the macroinitiator. Subsequently, the apparatus was inserted again in the glove box for monomer addition. The apparatus was equipped with a side ampoule with a ground joint and a constriction. The monomer amounts were added in order to obtain glycine or alanine to histidine molar ratios at 10%, 20%, and 40%. Trt-His-NCA (0.37g, 0.87 mmol) and Gly-NCA (0.0088 g, 0.088 mmol) were added for the polymer with 10% glycine to histidine molar ratio, while 0.01 g of Ala-NCA (0.088 mmol) (instead of Gly-NCA) was added to the polymer with a 10% molar ratio of alanine (instead of glycine) to histidine. The corresponding amounts for the polymers with 20% of glycine to histidine molar ratio were: 0.330 g (0.78 mmol) of Trt-His-NCA and 0.0188 g (0.19 mmol) of Gly-NCA, while 0.0214 g (0.19 mmol) of Ala-NCA (instead of Gly-NCA) was added to the polymer with a 20% alanine (instead of glycine) to histidine molar ratio. For the polymer with 40% glycine to histidine molar ratio, 0.310 gr (0.73 mmol) of Trt-His-NCA and 0.045 gr (0.45 mmol) of Gly-NCA were added, while 0.051 g (0.45 mmol) of Ala-NCA (instead of Gly-NCA) were added to the polymer with the 40% of alanine (instead of glycine) to histidine molar ratio.

The ground joint of the side ampoule of the apparatus containing the NCAs was subsequently connected to the vacuum line and, after evacuation, 5.0 mL of dry DMF were distilled. The reactor was removed from the vacuum line through heat sealing a constriction and the mixture of NCAs was dissolved. The rupture of the break-seal of the ampoule followed resulting in addition of the solution of the NCAs to the solution of the macroinitiator. After termination of the polymerization, the polymer was precipitated in diethyl ether and dried under high vacuum.

The polymer was suspended in CH_2_Cl_2_ (10 mL) and an equal volume of trifluoroacetic acid (TFA) was added. The polymer was completely dissolved and was left to be deprotected for 1 h at room temperature. An equimolar amount (with respect to the number of histidine monomeric units) of triethyl silane was added. The solution was distilled in the vacuum line in order to remove all solvents. The solid that remained was dissolved in water and was dialyzed against 2 liters of Milli-Q water with pH~3 (adjusted with a dilute aqueous solution of HCl) twice, twice in Milli-Q water with pH~9 (adjusted with a dilute aqueous solution of NaOH) and twice in pure Milli-Q water. Finally, the aqueous polymer solution was freeze dried to result in the corresponding polymer. The obtained polymer was 0.15 g for each polymerization.

### 2.6. Characterization

Size-exclusion chromatography. Size-exclusion chromatography (SEC) (Waters Corporation, Milford, MA, USA) was used to determine the *M*_n_ and *M*_w_/*M*_n_ values. It was composed of a Waters Breeze instrument equipped with a 2410 differential refractometer and a Precision PD 2020 two angle (15°, 90°) light scattering detector. A 0.10% TFA (*v*/*v*) solution of water/acetonitrile (60/40, *v*/*v*) at a flow rate of 0.8 mL/min at 35 °C was used as the carrier solvent. Three linear Waters hydrogel columns were employed. 

NMR spectroscopy. Proton nuclear magnetic resonance (^1^H NMR) spectroscopy (300 MHz) was performed using a Varian Unity Plus 300/54 spectrometer (Varian, Palo Alto, CA, USA) in deuterated dimethylformamide (DMSO).

FT-IR. Fourier transform infrared spectroscopy (FT-IR) measurements were performed with a Perkin Elmer Spectrum One instrument (Perkin Elmer, Waltham, MA, USA), in KBr pellets at room temperature, in the 450–4000 cm^−1^ range. 

Circular Dichroism. Circular Dichroism was performed with a Jasco J-815 model, featuring a Peltier model PTC-423S/15 thermo stabilizing system (Jasco Corporation, Tokyo, Japan). A 1 mm Quartz Suprasil cell was employed. Typical concentrations were about 7 × 10^−4^ g/ml.

Dynamic and static light scattering. Dynamic light scattering measurements were carried out with a Series 4700 Malvern system (Malvern Instruments Ltd., Worcestershire, UK) composed of a PCS5101 goniometer with a PCS7 stepper motor controller and a Cyonics variable power Ar^+^ laser, operating at 690 nm and with 10 mW power, a PCS8 temperature control unit, and a RR98 pump/filtering unit. Correlation function analysis included the cumulant method and the Contin software (Malvern Instruments Ltd., Worcestershire, UK). The correlation function was collected at 90°. Measurements were carried out five times for each sample and were averaged. Static light scattering measurements are taken with the same DLS instrument in a 30°–150°angular range, measured every 10 degrees. Measurements were carried out three times for each sample and were averaged. Toluene was used as the calibration standard. The dn/dc values were calculated from literature data [41]. The analysis of the results for the calculation of gyroscopic ratio was carried out using the 2nd order Guinier model. 

Zeta potential. The zeta potential of the synthesized polymers was measured by the technique of microelectrophoresis, using a Malvern Zetasizer 3000HSA instrument (Malvern Instruments Ltd., Worcestershire, UK) at room temperature at 633 nm. All the measurements were the average of at least three trials. The ζ-potential was calculated from the corresponding electrophoretic mobilities, μE, using the Henry’s correction of the Smoluchowski equation. 

Sample preparation. All measurements were carried out in isotonic Tris buffer (10 mM) with NaCl (150 mM) at pH = 7.4, in isotonic 2-(*N*-morpholino)ethanesulfonic acid (MES) buffer (10 mM) with NaCl (150 mM) at pH = 6.5, and in isotonic MES buffer (10 mM) with NaCl (150 mM) at pH = 5.0. Millipore water was thoroughly filtered with 0.1 μm filters and used directly for the preparation of the solutions. Initially, the polymers were dissolved in THF, followed by a dropwise addition of the appropriate amount of buffer solution, and removal of THF through heating and weighting the remained solution. In some cases, we added pure Milli-Q water in case that the weight was lower than the appropriate one. Typical concentrations were 5.0 mg of each polymer at 5.0 mL of the respective buffer. Sonication was performed when needed. No NaCl was used for the preparation of the samples measured with Zeta potential.

## 3. Results and Discussion

### 3.1. Synthesis

**Figure 1:**
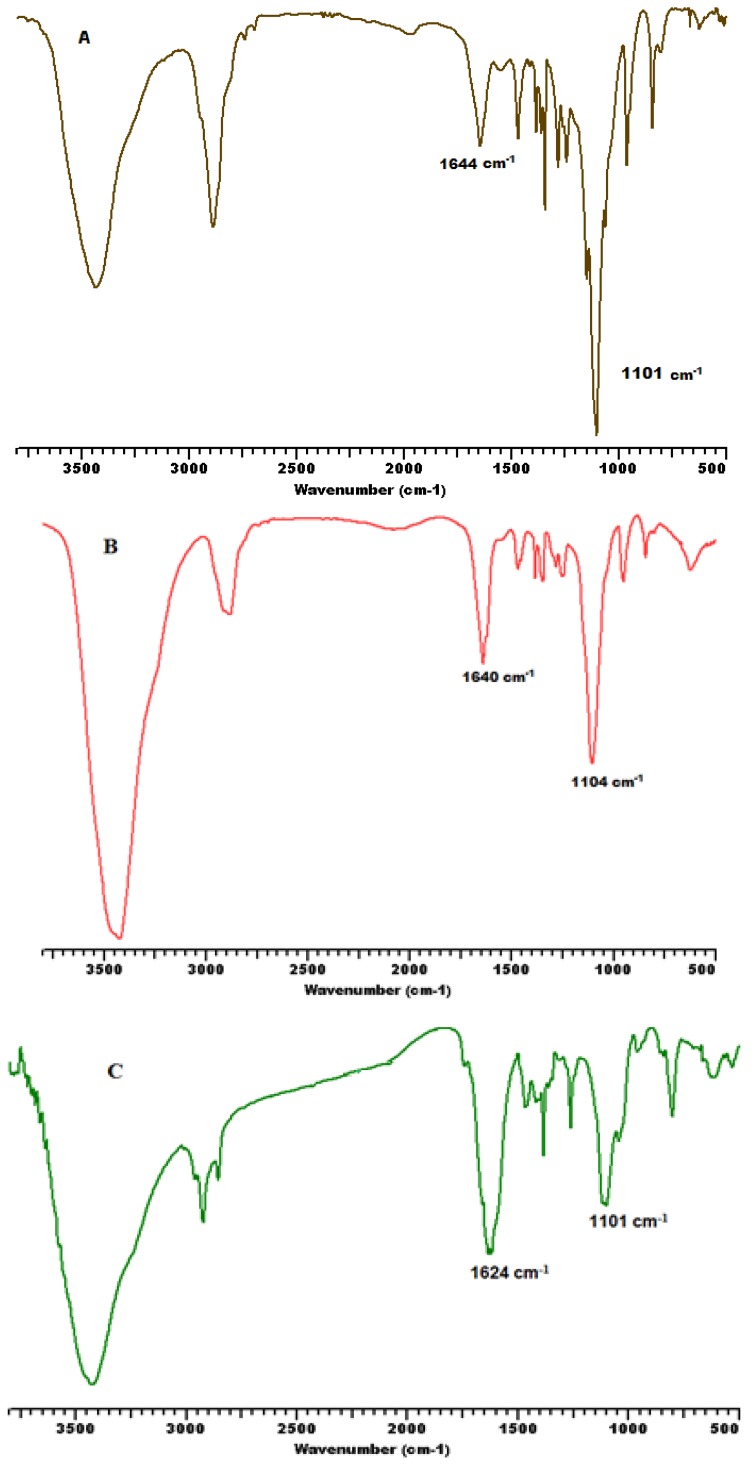
FT-IR spectra of *m*-PEO-*b*-poly(His-*co*-Gly) with (**A**) 10% glycine to histidine molar ratio; (**B**) 20% of glycine to histidine molar ratio; (**C**) 40% glycine to histidine molar ratio.

**Figure 2:**
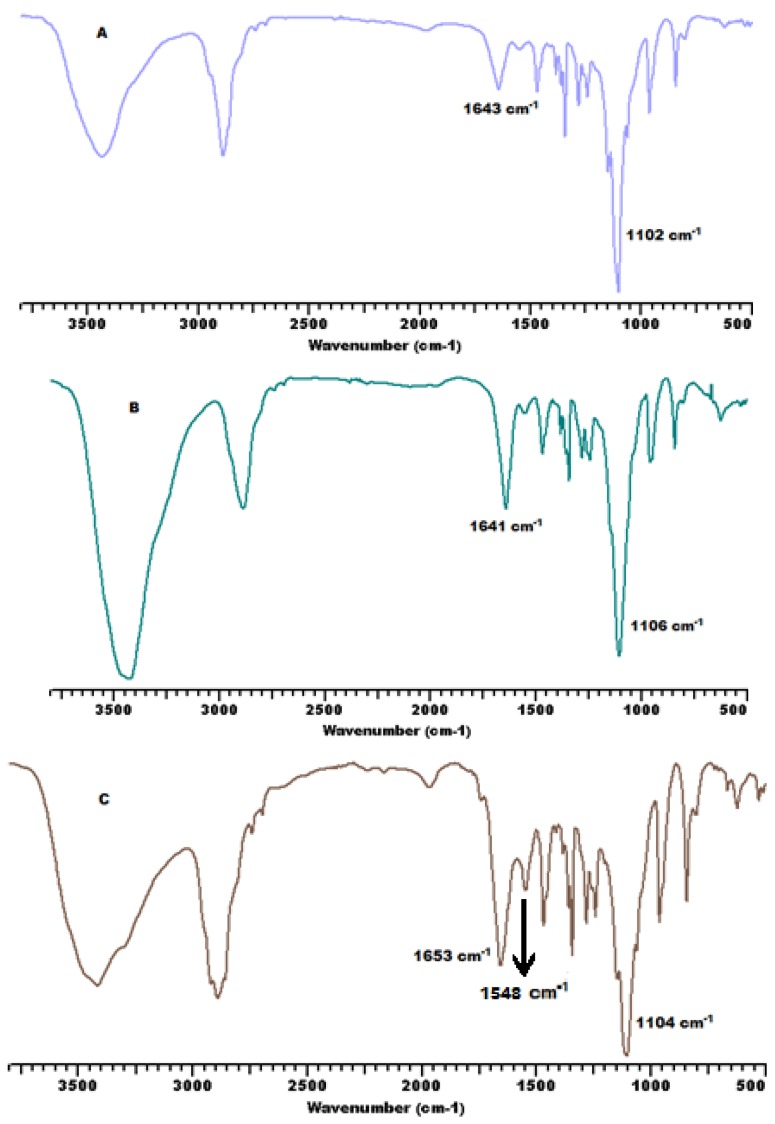
FT-IR spectra of *m*-PEO-*b*-poly(His-*co*-Ala) with (**A**) 10% alanine to histidine molar ratio (**B**) 20% alanine to histidine molar ratio (**C**) 40% alanine to histidine molar ratio.

**Figure 3:**
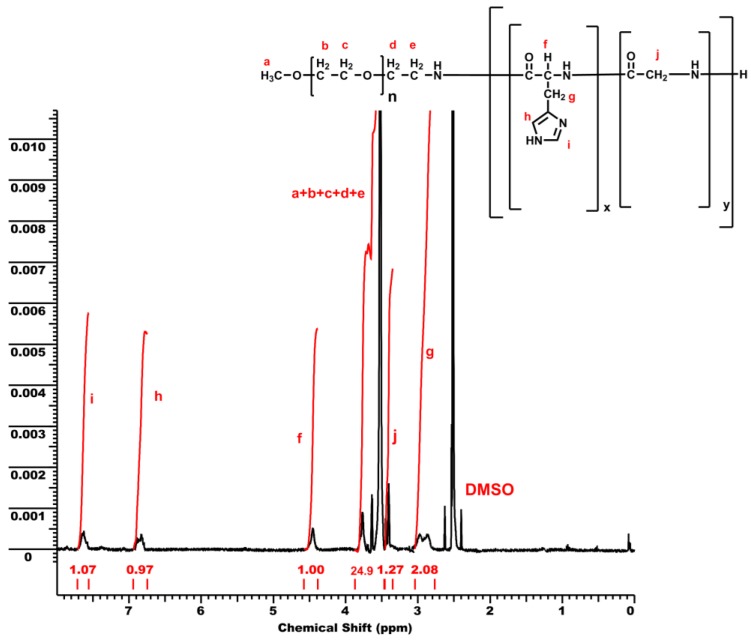
^1^H NMR spectrum of *m*-PEO-*b*-poly(His-*co*-Gly) with 40% of glycine to histidine molar ratio.

**Figure 4:**
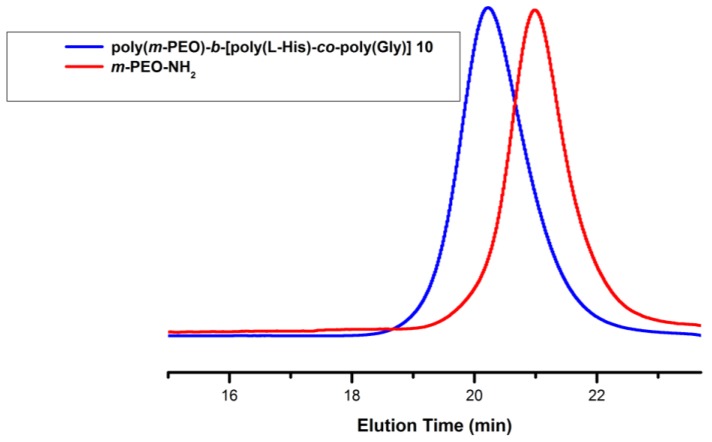
SEC eluogram of *m*-PEO-*b*-poly(His-*co*-Gly) with 10% glycine to histidine molar ratio.

**Figure 5:**
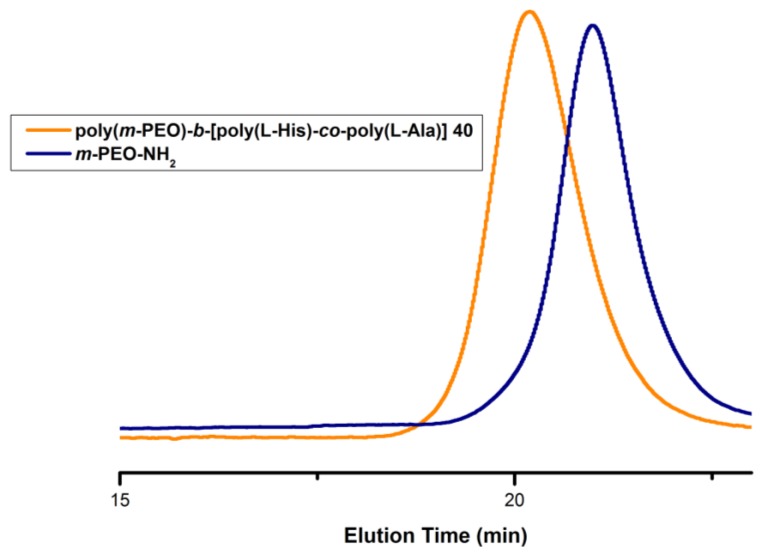
SEC eluogram of *m*-PEO-*b*-poly(His-*co*-Ala) with 40% alanine to histidine molar ratio.

The synthetic routes followed for the preparation of the hybrid-copolypeptides *m*-PEO-*b*-poly(His-*co*-Gly) and *m*-PEO-*b*-poly(His-*co*-Ala) are depicted in Scheme 2 and Scheme 3, respectively. The hybrid-polypeptides were synthesized in such a way that the total molecular weight of the polypeptidic block would be close to 6.0 × 10^3^ g/mol. The high purity of the synthesized NCAs is crucial for maintaining the living nature of the polymerization as well the complete monomer consumption. If traces of protic impurities remain in NCAs, the polymerization will slow down or will be completely terminated, due to termination of the living active ends. In every polymerization, the macroinitiator (*m*-PEO-NH_2_) was dissolved in purified, dried benzene and then the benzene was distilled off to dryness. This step was necessary in order to remove residual water in PEO, which can trigger NCA polymerization. The CO_2_ produced from the polymerization was also periodically removed by degassing under high vacuum. All the copolymerizations showed a lack of heterogeneity during the polymerization in contrast to the homopolymerization of histidine NCA for the synthesis of *m*-PEO-*b*-poly(His) (presented in a previous work [18]), which was heterogeneous as the solution turned turbid during the polymerization [25]. This is a strong indication that the monomeric units of glycine and alanine are randomly distributed in the polypeptide block. The polymerizations lasted 7 days, and the consumption of the monomers was simultaneously monitored by FT-IR through the removal of an aliquot of the solution in the glove box. The disappearance of the peaks at 1780 and 1850 cm^−1^, which correspond to the cyclic NCAs, and the appearance of the peak at 1650 cm^−1^, corresponding to the peptide bond during the formation of the polymer, verified polymerization completion. The successful removal of the Trt protection group of the histidine residues was confirmed with FT-IR, as no peaks were found at 700 and 750 cm^−1^ (Figure 1 and Figure 2). The peak at 1104 cm^−1^ is due to the –C–O– bond of the macroinitiator (PEO). Additionally, no indication for the Trt protecting group was found by ^1^H NMR of the final polymers. The ^1^H NMR spectrum is provided in Figure 3. The SEC eluograms of the PEO-*b*-P(His-*co*-Gly) and PEO-*b*-P(His-*co*-Ala) are shown in Figure 4 and Figure 5, respectively. A high degree of molecular and compositional homogeneity of the copolymers are confirmed by the low polydispersity indices. The composition of the blocks of the His and either Gly or Ala random copolypeptides, was estimated from the NMR spectra and found to be identical with the NCA feed ratio. The molecular characteristics of the synthesized polymers are provided in Table 1.

### 3.2. Secondary Structure

The conformational analysis of each diblock polypeptide was performed using FT-IR in solid state after lyophilization and CD in the solution state in Milli-Q water. All the synthesized polypeptides exhibit an intense amide I band, which is governed mainly by the stretching vibrations of the C=O group. In the dry state, the polypeptides with the glycine residues at 10% and 20% molar ratios, as well as the polypeptides with alanine residues at 10% and 20% molar ratios, display an intense amide I band around 1640 cm^−1^. This vibration is assigned to random coil conformation. The adoption of the random coil motifs by these polypeptides in the solid state renders the formation of an intramolecular network difficult. The polypeptide with the glycine residues at 40% molar ratio displays a peak at 1624 cm^−1^, which suggests the *β*-sheet formation. It is well-known that Poly(Gly), depending on the casting conditions, can very often exist in *β*-sheet structures, which influence the segmental mobility [28]. The polypeptide with the alanine residues at 40% molar ratio has two peaks. One at 1653 cm^−1^, corresponding to amide I band and one at 1548 cm^−1^, corresponding to amide II band. Both vibrations are attributed to *α*-helix formation. Gitsas et al. suggested that although pure Poly(Ala) stabilizes both *α*-helices and *β*-sheets, *α*-helices are thermodynamically more stable in the copolypeptides [42]. The change of the secondary structure of the polypeptides in the solid state after lyophilization, as the monomeric units of either glycine or alanine increase and the respective monomeric units of histidine decrease, constitutes an additional indication that the glycine and alanine residues are distributed randomly along the PHis chain.

**Figure 6:**
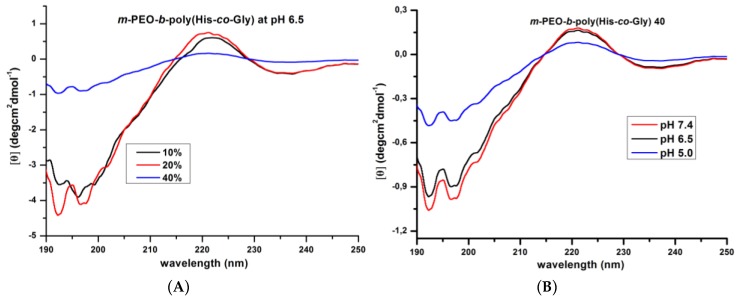
(**A**) CD of *m*-PEO-*b*-poly(His-*co*-Gly) with different Gly to His molar ratios at pH 6.5; (**B**) CD of *m*-PEO-*b*-poly(His-*co*-Gly) with 40% molar ratio of Gly residue to His residue as a function of pH.

**Figure 7:**
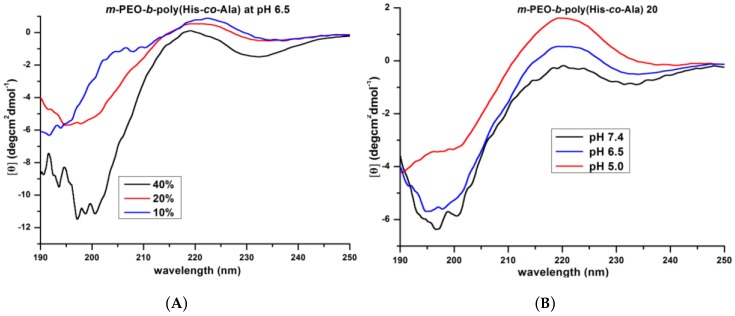
(**A**) CD of *m*-PEO-*b*-poly(His-*co*-Ala) with different Ala to His molar ratios at pH 6.5; (**B**) CD of *m*-PEO-*b*-poly(His-*co*-Ala) with 20% molar ratio of Ala residue to His residue as a function of pH.

Circular dichroism spectroscopy was used to reveal the secondary structure of these polypeptides and particularly to elucidate how the randomly distributed residues of glycine and alanine influence the conformation of PHis in water (Figure 6 and Figure 7). CD measurements were performed for all the synthesized polymers at pH values 7.4, 6.5 and 5.0 and also at temperatures 25 °C, 37 °C and 41 °C. Since glycine is achiral, the monomeric units of glycine do not participate in the spectra obtained for the polypeptides of the type *m*-PEO-*b*-poly(His-*co*-Gly). Only PHis contributes and governs the secondary structures. All of the glycine containing diblocks exhibited a broad negative trough below 200 nm and a positive peak above 220 nm. These results are in agreement with the random coil conformation, which is adopted for all the diblocks with the different molar ratios of Gly to His residue. The same motif is maintained at every pH. It can also be observed that the θ magnitude for the diblock with 40% molar ratio of glycine residue to histidine residue decreased due to the reduced monomeric units of histidine. The same conformation was obtained for all the polymers including alanine moieties, despite the fact that alanine is not achiral. Specifically, all the polypeptides of the type PEO-*b*-poly(His-*co*-Ala) provide a broad negative peak below 200 nm and a positive one above 220 nm, which can be assigned to the random coil conformation. This conformation is also maintained at every pH. All the synthesized polymers were examined with CD as a function of temperature showing no change in their secondary structure.

These results are comparable with CD spectroscopy studies formerly carried out by our group on similar systems [25]. In the case of the diblock *m*-PEO-*b*-poly(His), the conformation is almost identical at pH 5.0, as the random coil conformation is also preferred by the polymer. The protonated moieties of histidine undergo repulsion, which is a deterrent factor for the formation of an ordered structure. As the pH value increased the diblock *m*-PEO-*b*-poly(His) presented a different secondary structure as *β*-sheet conformation developed. The synthesized polymers of the current work do not seem to follow this trend, probably because the hydrophobic monomeric units of glycine or alanine do not allow the histidine monomeric units to form organized structures. The choice of an appropriate hydrophobic amino acid to be randomly distributed among the PHis chain is crucial for the control of the properties of these systems. In the same work [25], it was found that leucine monomeric units direct the conformation of the diblock PEO-*b*-poly(His-*co*-Leu) to *α*-helix at 40% molar ratio; however, this did not happen in this work in the case glycine or alanine monomeric units.

### 3.3. Aggregation Properties

DLS was utilized to elucidate the aggregation behavior of the synthesized polymers. PHis has the ability to become hydrophilic and interact with water at lower pH due to its protonation [43]. The participation of hydrophobic monomeric units randomly distributed within the PHis chain can change the aggregation phenomena having, as a result, the increase in the dimensions of the aggregates [25]. Investigation of the pH-dependence of the aggregate dimensions involved comparing the hydrodynamic radii (*R*_*H*_) values to those observed at the same temperature at three different pH values (7.4, 6.5, 5.0) for each different sample. The results from DLS are illustrated in Table 2. It should be mentioned that at pH 7.4 the measurements were performed after filtration of the samples, while no filtration occurred for the samples at different pH. It is obvious that the general trend is an increase in the dimensions of the aggregates as the pH decreases. This is due to the partial protonation of PHis, which renders the aggregates more hydrophilic leading them to swell. The protonation of the histidine residues in the polypeptide block converts the hydrophobic domain to hydrophilic at lower pH. Probably, this phenomenon is related to the p*K*_a_ of the samples, which plays a crucial role for the aggregation properties. For the diblocks *m*-PEO-*b*-poly(His-*co*-Gly), the increase of the molar ratio of the glycine to histidine residue has, as a result, an increase of the R_H_ at every pH. Additionally, as the pH value decreases for all the diblocks containing glycine monomeric units, the KCounts of the scattering light are also lower, suggesting partial disruption of the aggregates. For the diblocks *m*-PEO-*b*-poly(His-*co*-Ala), the increase of the alanine to histidine molar ratio follows no particular trend. Furthermore, all the alanine containing aggregates are swollen upon lowering the pH. The reduction of the KCounts of the scattering light is also observed with the decrease in pH for all the samples containing alanine monomeric units. The large size of polymeric structures (over 100 nm) is most likely due to the formation of secondary aggregates that are clusters of single aggregates. The participation of the hydrophobic monomeric units of glycine and alanine into the PHis chain increases the dimensions of the structures compared to the structures of *m*-PEO-*b*-poly(His) [25]. Static light scattering measurements were also performed using the same conditions. The results are presented in Table 3. The ratio of the radii of gyration (*R*_G_) to *R*_H_ (*R*_G_/*R*_H_ ratio) depends on the morphology of the aggregates formed. *R*_G_/*R*_H_ values close to 1 can be attributed to the formation of vesicles, whereas smaller values are generally attributed to spherical micelles (theoretically 0.78) [44]. All the samples at pH value 7.4 have a tentative micellar morphology, while as the pH value decreases the *R*_G_/*R*_H_ ratio also decreases. Note that these *R*_G_/*R*_H_ ratio values are lower than the value known for a hard sphere (*R*_G_/*R*_H_ = 0.78). At lower pH values, the dimensions of the aggregates are much higher than 100 nm, and the obtained *R*_G_ values are apparent.

**Figure 8:**
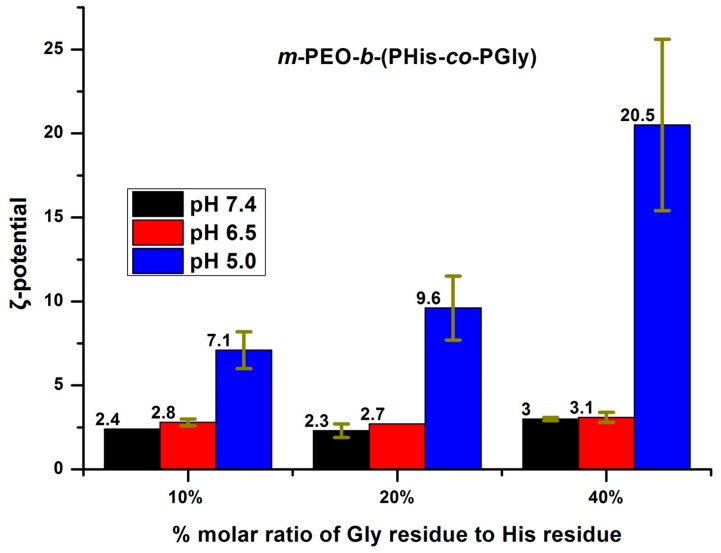
Zeta potential measurements of the synthesized polypeptides containing glycine residues as a function of pH and glycine to histidine residue molar ratio percentage.

**Figure 9:**
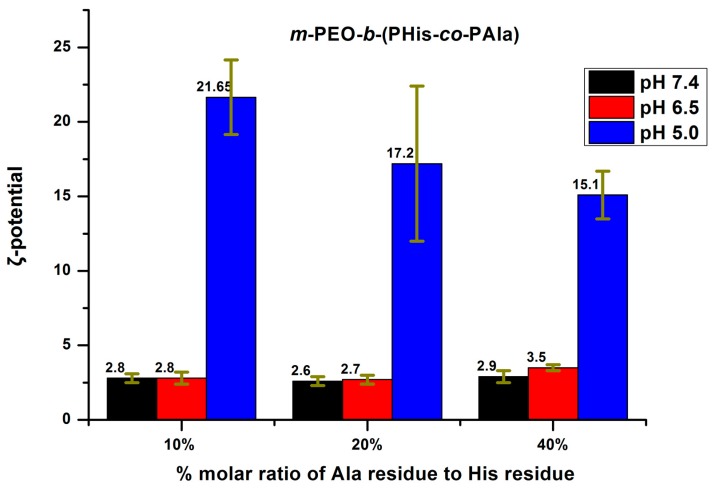
Zeta potential measurements of the synthesized polypeptides containing alanine residues as a function of pH and of alanine to histidine molar ratio percentage.

The dissolution of the synthesized polymers in aqueous media, directs the PEO blocks towards the aqueous phase, while the hydrophobic polypeptidic domain of the diblocks is physically entangled in the inner matrix. The formed hydrophilic PEO shell presents no surface charges, which offers “stealth” properties to the nanostructures against the reticuloendothelial system. As a result, they become invulnerable to macrophages. The results of zeta potential measurements are presented in Figure 8 and Figure 9. The utilization of this method for all the samples for both types of polypeptides at pH 7.4 and 6.5 revealed that their surface charges are close to 0 mV. These results meet the condition of zero surface charges for the characterization of the nanostructures as “stealth”. The pH responsivity of the nanoparticles is confirmed by the increase of the surface charges of all the polymers at pH 5.0. An increase of zeta potential for the measurements of all samples at pH 5.0 is observed. The partial protonation of the PHis in the polypeptidic block due to the lower pH values is the main reason for the swelling of the nanostructures. One of the consequences of this change is the increase of the surface charges of these formations to more positive values, as the histidine residues are no longer entangled in the inner matrix, but can extend to the outer periphery of the aggregates and interact with water due to the increase of their hydrophilicity. Surprisingly, it was found that the polymer containing 40% of glycine protonates more strongly than those with 10% and 20% glycine. This can be attributed to the likely formation of vesicular structures at pH = 7.4, due to the *R*_G_/*R*_H_ obtained close to 1, while the polymers with 10% and 20% probably form core-shell micelles. 

## 4. Conclusions

Employing high vacuum techniques and the ROP of *α*-amino acid NCAs, we successfully synthesized a series of novel diblock polypeptides of the type *m*-PEO-*b*-poly(His-*co*-Gly) with glycine to histidine molar ratios at 10%, 20%, 40% and of the type *m*-PEO-*b*-poly(His-*co*-Ala) with alanine to histidine molar ratios at 10%, 20%, 40%. The molecular characterization results for all synthesized terpolymers show that the polymers had a high degree of molecular and compositional homogeneity. The study of their secondary structure in solid state revealed that the polypeptide with 40% molar ratios of glycine to histidine adopts *β*-sheet formation, while the polypeptide with 40% molar ratios of alanine to histidine adopts *α*-helix formation. All other synthesized polymers adopt random coil formation. The random coil conformation was revealed for all the synthesized polymers in water. Studies on the self-assembly of these terpolymers in aqueous solutions showed that they form aggregates with the general trend of the dimensions to increase as the pH decreases. All the samples at pH 7.4 showed a tentative micellar morphology, with the exception of the 40% Gly-containing polymer that probably exhibits a vesicular structure. Zeta potential experiments revealed that the surface charges of the nanostructures of all the synthesized polymers at pH 5.0 are larger and positive in comparison with the surface charges of the nanostructures at pH 7.4 and pH 6.5, which is almost zero. This shows that at pH = 7.4 and 6.5 the PEO remain in the outer periphery, while at pH = 5.0 the strong protonation of PHis results in more swollen chains that reach the shell of the nanoparticle. The study of the properties of the synthesized diblocks revealed that based on the composition and the nature of the hydrophobic amino acid along the PHis chain, it is possible not only to alter the aggregation morphology of the nanoconstructs but also their pH-responsiveness. It was shown that at lower molar ratios of the hydrophobic amino acids Gly and Ala along the PHis chain, PHis becomes very hydrophobic and a significant decrease in pH is required to disrupt the aggregates, as compared to the p*K*_a_ of 6.5 of pure PHis. This could be an interesting property for the encapsulation of hydrophobic drugs within the aggregate core to be released only under lower pH conditions, and therefore remaining stable during circulation within the blood compartment to be delivered only upon internalization within the cells.

## Figures and Tables

**Table 1:** Molecular characteristics of the synthesized polymers.

Polymer	*M*_n_ 1st Block × 10^−3 a^	*M*_n_ 2nd Block × 10^−3 b^	Total *M*_n_ × 10^−3 c^	I ^d^	Composition % (*w*/*w*) ^e^
*m*-PEO-*b*-poly(His-*co*-Gly) 10	9.95	6.20	16.1	1.13	PEO/His/Gly: 62/36/2
*m*-PEO-*b*-poly(His-*co*-Gly) 20	9.95	5.75	15.6	1.11	PEO/His/Gly: 62.1/34/3.9
*m*-PEO-*b*-poly(His-*co*-Gly) 40	9.95	6.10	15.9	1.15	PEO/His/Gly: 62/30/8
*m*-PEO-*b*-poly(His-*co*-Ala) 10	9.95	6.30	16.1	1.10	PEO/His/Ala: 62.8/35/2.2
*m*-PEO-*b*-poly(His-*co*-Ala) 20	9.95	6.0	16.1	1.09	PEO/His/Ala: 62.6/33/4.4
*m*-PEO-*b*-poly (His-*co*-Ala) 40	9.95	6.30	16.2	1.14	PEO/His/Ala: 62.7/27.5/9.8

**^a^** Obtained by SEC-Two Angles Laser Light Scattering (TALLS). **^b^** Obtained by the difference between the molecular weight of the first block and the molecular weight of the diblock obtained by SEC-TALLS. **^c^** Molecular weight of the final diblock obtained by SEC-TALLS. **^d^** Polydispersity index obtained by SEC. **^e^** Obtained by ^1^H NMR spectra.

**Table 2:** DLS results for the synthesized polymers at 25 °C and 90°.

Sample	pH = 7.4 (Filtration)			pH = 6.5			pH = 5.0		
	*R*_H_ (nm)	Poly	KCounts	*R*_H_ (nm)	Poly	KCounts	*R*_H_ (nm)	Poly	KCounts
*m*-PEO-*b*-poly(His-*co*-Gly) 10	92.6	0.34	812	112.4	0.252	624	161.2	0.40	521
*m*-PEO-*b*-poly(His-*co*-Gly) 20	113.1	0.24	873	128.1	0.21	723	223	0.44	432
*m*-PEO-*b*-poly(His-*co*-Gly) 40	120.6	0.293	908	144.0	0.452	827	673.9	0.505	567
*m*-PEO-*b*-poly(His-*co*-Ala) 10	96.5	0.309	582	132	0.462	430	143.3	0.394	307
*m*-PEO-*b*-poly(His-*co*-Ala) 20	118.2	0.237	1013	163.7	0.416	908	165.3	0.36	497
*m*-PEO-*b*-poly(His-*co*-Ala) 40	111.5	0.23	761	121	0.309	714	(1) 175.5 (2) 1005	0.467	647

**Table 3:** *R*_G_/*R*_H_ ratio from SLS/DLS measurements for samples of the synthesized polymers at 25 °C.

Sample	pH = 7.4 (Filtration)			pH = 6.5			pH = 5.0		
	*R*_G_^a^	*R*_H(0)_^b^	*R*_G_/*R*_H(0)_	*R*_G_^a^	*R*_H(0)_^b^	*R*_G_/*R*_H(0)_	*R*_G_^a^	*R*_H(0)_^b^	*R*_G_/*R*_H(0)_
*m*-PEO-*b*-poly(His-*co*-Gly) 10	126.1	180.7	0.69	142.6	288.4	0.49	197.1	898.6	0.22
*m*-PEO-*b*-poly(His-*co*-Gly) 20	137.3	222.2	0.62	194.1	886.7	0.22	197.8	479.1	0.42
*m*-PEO-*b*-poly(His-*co*-Gly) 40	152.6	191.1	0.95	184.5	347.7	0.53	221.7	743.7	0.30
*m*-PEO-*b*-poly(His-*co*-Ala) 10	119.9	181.9	0.65	186.2	883.1	0.21	199	400.4	0.49
*m*-PEO-*b*-poly(His-*co*-Ala) 20	123.1	153.9	0.80	188.6	860.6	0.21	194.4	483.6	0.40
*m*-PEO-*b*-poly(His-*co*-Ala) 40	114.3	154.4	0.74	158.5	326.2	0.48	211.7	825.8	0.25

^a^ Obtained by SLS. ^b^ Values obtained after extrapolation at 0 degree.
